# Targeted Therapies in Myelofibrosis: Present Landscape, Ongoing Studies, and Future Perspectives

**DOI:** 10.1002/ajh.27658

**Published:** 2025-03-10

**Authors:** Giuseppe G. Loscocco, Paola Guglielmelli

**Affiliations:** ^1^ Department of Experimental and Clinical Medicine, CRIMM, Center of Research and Innovation of Myeloproliferative Neoplasms, Azienda Ospedaliero‐ Universitaria Careggi University of Florence Florence Italy; ^2^ Division of Hematology Mayo Clinic Rochester Minnesota USA

**Keywords:** disease modification, JAK inhibitors, JAK–STAT pathway, myelofibrosis, targeted therapies

## Abstract

Myelofibrosis (MF) is a myeloproliferative neoplasm that is accompanied by driver *JAK2*, *CALR*, or *MPL* mutations in more than 90% of cases, leading to constitutive activation of the JAK–STAT pathway. MF is a multifaceted disease characterized by trilineage myeloid proliferation with prominent megakaryocyte atypia and bone marrow fibrosis, as well as splenomegaly, constitutional symptoms, ineffective erythropoiesis, extramedullary hematopoiesis, and a risk of leukemic progression and shortened survival. Therapy can range from observation alone in lower‐risk and asymptomatic patients to allogeneic hematopoietic stem cell transplantation, which is the only potentially curative treatment capable of prolonging survival, although burdened by significant morbidity and mortality. The discovery of the *JAK2* V617F mutation prompted the development of JAK inhibitors (JAKi) including the first‐in‐class JAK1/JAK2 inhibitor ruxolitinib and subsequent approval of fedratinib, pacritinib, and momelotinib. The latter has shown erythropoietic benefits by suppressing hepcidin expression via activin A receptor type 1 (ACVR1) inhibition, as well as reducing splenomegaly and symptoms. However, the current JAKi behave as anti‐inflammatory drugs without a major impact on survival or disease progression. A better understanding of the genetics, mechanisms of fibrosis, cytopenia, and the role of inflammatory cytokines has led to the development of numerous therapeutic agents that target epigenetic regulation, signaling, telomerase, cell cycle, and apoptosis, nuclear export, and pro‐fibrotic cytokines. Selective JAK2 V617F inhibitors and targeting of mutant CALR by immunotherapy are the most intriguing and promising approaches. This review focuses on approved and experimental treatments for MF, highlighting their biological background.

## 1 Introduction

Myelofibrosis (MF) is one of the Philadelphia‐negative myeloproliferative neoplasms (MPN) along with polycythemia vera (PV) and essential thrombocythemia (ET) and can arise as *de novo* disease, referred to as primary MF (PMF), or secondary (SMF) to PV and ET (post‐PV/ET MF) [1]. The 2016 revision to the World Health Organization (WHO) classification for myeloid malignancies [2] formally subcategorized PMF into “prefibrotic/early stage MF” and “overt fibrotic stage MF” This PMF sub‐classification has been retained and implemented by specific diagnostic criteria in the latest 2022 WHO classification revision [3] and also in the International Consensus classification of myeloid neoplasms [4]. The latter also defines diagnostic criteria for post‐PV/ET MF, adopting the precedent criteria published by the International Working group for MPN Research and Treatment (IWG‐MRT) [5]. Overall, MF diagnosis relies on histopathological and molecular criteria. The bone marrow (BM) is hypercellular or hypocellular, and megakaryocytes are numerous and arranged in tight clusters showing pleomorphism and dysplastic features. Typically, varying degrees of fibrosis are reported (grade 0‐1 in pre fibrotic MF and grade 2–3 in overt fibrotic MF). Driver mutations involving *JAK2*, *CALR*, or *MPL* in approximately 90% of patients mediate constitutive JAK‐STAT signaling which, along with additional alterations (*ASXL1*, *SRSF2*, *EZH2*, *IDH1*/2, *U2AF1* mutations), play a crucial role in disease pathogenesis and also in attributing prognosis [6]. Clinical manifestations in MF include hepatosplenomegaly, constitutional symptoms (e.g., fatigue, night sweats, fever), bone pain, cachexia, pruritus, thrombosis, and bleeding, related to aberrant cytokine production by clonal cells and host immune reaction [7, 8]. Ineffective erythropoiesis and hepatosplenic extramedullary hematopoiesis are the main causes of anemia and organomegaly, respectively. Although the most frequent cause of death is disease progression that occurs in approximately 20% of patients, patients are at high risk for other competing causes of morbidity and mortality including cardiovascular events and consequences of cytopenias including infection or bleeding [9]. The considerable heterogeneity of MF translates into a wide range of outcomes, with overall survival (OS) spanning a few years to decades. Given the clinical heterogeneity, risk stratification for optimal individual management is crucial [10]. In this regard, the main contemporary prognostic systems for PMF include the Molecular Enhanced International Prognostic Score Systems (three‐tiered MIPSS70 [11], four‐tiered MIPSS70‐plus [11], and five‐tiered MIPSS70‐plus 2.0 [12]). The latter includes *U2AF1* Q157 as a high molecular risk variant along with *ASXL1*, *EZH2*, *IDH1*, *IDH2*, and *SRSF2* mutations, a new sex‐ and severity‐adjusted hemoglobin thresholds for anemia, and a refined three‐tiered cytogenetic risk distribution with the introduction of a very high risk (VHR) group including patients with single/multiple abnormalities of −7, i(17q), inv (3)/3q21, 12p‐/12p11.2, 11q‐/11q23, or other autosomal trisomies not including +8/+9. A four‐tiered Myelofibrosis Secondary to PV and ET‐Prognostic Model (MYSEC‐PM) was specifically developed for secondary SMF, including older age, hemoglobin < 11 g/dL, circulating blasts ≥ 3%, platelet count < 150 ×10^9^/L, constitutional symptoms, and *CALR* mutational status as variables [13]. Despite the above‐mentioned advances in prognostic stratification of patients with both PMF and SMF, the most widely used score, especially in the subset of clinical trials, is the four‐tiered Dynamic International Prognostic Scoring System (DIPSS) including age > 65 years, hemoglobin < 10 g/dL, leukocyte count > 25 × 10^9^/L, circulating blasts ≥ 1%, and constitutional symptoms [14].

Treatment options for MF range from supportive care (i.e., transfusional support) to allogeneic hematopoietic stem cell transplantation (allo‐HSCT), which, at the moment, is the only potentially curative treatment, although it is burdened by not negligible toxicity and mortality [15]. In recent years, as drug therapies, JAK inhibitors (JAKi) played a leading role, with the first‐in‐class ruxolitinib paving the way for others (e.g., fedratinib, pacritinib and momelotinib) with the potential to reduce symptoms and spleen size and improve quality of life, unfortunately without modifying the natural history of the disease [16].

Therefore, a number of studies with novel treatments for JAK inhibitor‐ineligible or relapsed/refractory patients are ongoing. Starting from MF pathophysiology and highlighting targetable pathways beyond JAK –STAT, the current review provides an overview of current and experimental treatments both as monotherapy and combinations. Anemia‐focused therapies will be discussed in depth in another review paper of the series.

## 2 Pathophysiology and Potential Targets for Therapies

### 2.1 Molecular Characterization

A new era on MPN started in 2005 with the discovery of the *JAK2V617F* mutation by four independent groups in nearly all patients with PV and in 50%–60% of patients with ET and MF [17, 18, 19, 20]. Since then, it has been widely recognized that constitutive activation of JAK‐STAT signaling plays a pivotal role in the pathogenesis of MPN with or without the presence of the *JAK2* V617F mutation. Subsequent studies focused on characterizing genetic alterations in *JAK2* wild‐type patients led to the identification in 2006 of mutations in the thrombopoietin (TPO) receptor *MPL*, which specifically regulates megakaryopoiesis and platelet production through the JAK‐STAT pathway. Mutations in *MPL* are found in 3%–8% of ET and PMF and consist of gain‐of‐function variants at tryptophan 515 (W515) in exon 10 [21]. The most common point mutations are W515L and W515K; other rare variants include W515R, W515A, and W515G [22]. In 2007, *JAK2* exon 12 mutations, particularly in‐frame insertions or deletions between codons 536 and 544, were discovered in *JAK2* V617F‐negative PV patients [23, 24]; *JAK2* exon 12 mutations were also detected in post‐PV MF cases, not in ET, post‐ET MF, and PMF cases [23]. In 2013, two independent groups described a novel mutation in most *JAK2* and *MPL* wild‐type patients [25, 26]. By applying whole exome sequencing, they identified somatic mutations in *CALR* encoding for calreticulin, a highly conserved endoplasmic reticulum lectin chaperone that assists in the folding of glycoprotein substrates and maintenance of cell calcium homeostasis given its negatively charged C‐terminus [27]. To date, more than 50 different mutations in *CALR* exon 9 have been described in approximately 20%–30% of ET and PMF cases [25, 26]. The two most frequent *CALR* mutations, accounting for approximately 80% of all mutations, include a type‐1 52‐bp deletion (c.1092_1143del; p.L367fs*46) and a type‐2 5‐bp insertion (c.1154_1155insTTGTC; p.K385fs*47). Other atypical mutations are grouped as type 1‐ or type 2‐like in relation to predicted helical secondary structure or the number of calcium‐binding amino acids remaining in the novel C‐terminus [28]. Over the past 10 years, extensive work unraveled oncogenetic mechanisms by mutant *CALR* both in cellular and murine models. Overall, mutant CALR activates MPL resulting in constitutive activation of JAK/STAT signaling [29, 30, 31, 32, 33]. Of interest, the cell surface expression of the mutated CALR–MPL complex is a mandatory feature for MPL and JAK‐STAT signaling activation [29, 30, 31, 32, 33]. Recently, high‐throughput next‐generation sequencing (NGS) approaches identified additional somatic mutations with a diagnostic, prognostic, and therapeutic value. At least one additional mutation can be found in more than 80% of patients. These mutations are not disease‐specific but also occur in other myeloid malignancies including acute myeloid leukemia (AML) [34], myelodysplastic syndromes (MDS) [35], and MDS/MPN [36], as well as in healthy individuals in the context of age‐related clonal hematopoiesis (ARCH/CHIP) [37]. Additional genomic abnormalities affect genes involved in DNA methylation (*TET2*, *DNMT3A*, *IDH1*, and *IDH2*), histone modification (*ASXL1*, *EZH2*), mRNA splicing (*SFRB1*, *SRSF2*, *U2AF1*, and *ZRSR2*), signaling pathways (*LNK*/*SH2B3*, *CBL*, *NRAS*, *KRAS*, and *PTPN11*), and transcription factors (*SETBP1*, *RUNX1*, *NFE2*, *TP53*, and *PPM1D*) [10]. Around 10% of patients with PMF are negative for all three driver mutations and are referred to as “triple‐negative” Of interest, at least 10% of PMF patients harbor mutations outside of canonical *MPL* and *JAK2* hotspots leading to a constitutive activation of the JAK‐STAT signaling. Non‐canonical *MPL* mutations include T119I, S204F/P, and E230G in the extracellular domain and Y591D/N in the intracellular domain [38], whereas non‐canonical *JAK2* mutations include V625F, F556V, R683G, and E627A [39]. Overall, gene expression profiling studies confirmed that JAK‐STAT activation occurs in all MF patients regardless of *JAK2* V617F mutational status [40]. Recently, *SETBP1* mutations were detected in around 20% of triple‐negative PMF cases as an early genetic event, characterized by a dismal outcome [41]. In addition, RAS pathway mutations significantly impact the progression and survival outcomes in MF by influencing resistance to JAK inhibitors and promoting clonal evolution through enhanced proliferation and survival of malignant hematopoietic cells [42].

### 2.2 Bone Marrow Fibrosis, Inflammation, and Microenvironment

The JAK‐STAT activation partially explains the complex pathophysiology of MF since the same genetic alterations are reported also in other myeloid neoplasms. The deposition of reticulin and collagen fibers is one of the MF hallmarks, and it is considered a reactive process mediated by mutated hematopoietic stem cells (HSC), contributing to an impaired microenvironment favoring malignant over normal hematopoiesis. Moreover, increased expression of inflammatory cytokines, lysyl oxidase, and transforming growth factor‐β (TGF‐β), mainly driven by aberrant megakaryocytes, has been implicated in the pathogenesis of fibrosis [43]. The process of megakaryocytic hyperplasia and subsequent fibrosis was clarified by in vitro studies performed on CD34 + cells isolated from MF patients. In a seminal study by Ciurea et al., a TPO‐independent increased propensity of CD34 + cells to generate megakaryocytes was demonstrated, along with impaired apoptosis due to overexpression of anti‐apoptotic proteins (Bcl‐xL) [44]. In addition, higher levels of TGF‐β and active matrix metalloproteinase‐9 (MMP9) secreted by MF CD61+ cells were detected [44]. TGF‐β is a pleiotropic cytokine that stimulates fibroblasts to produce extracellular matrix and occurs in three isoforms: TGFβ‐1, TGFβ‐2 and TGFβ‐3. The first is the most relevant of all these isoforms, secreted mainly by megakaryocytes [45, 46]. The pro‐fibrotic effect of TGFβ‐1 is twofold: on the one hand, it increases the synthesis of types I, III, and IV collagen, as well as the deposition of fibronectin, proteoglycans, and tenascin [47]; on the other hand, it decreases matrix degradation [48]. TGFβ‐1 seems to be crucial for marrow fibrosis development in mouse models by inducing mesenchymal stem cell proliferation and transition to myofibroblasts and osteoblasts [49].

Another important determinant in the pathogenesis of MF is the role of pro‐inflammatory and pro‐fibrotic cytokines. Whether mutant hematopoietic cells can trigger inflammation, or whether the inflammatory environment can be responsible for genotoxic stress and thus induce malignancy, is still debated; possibly, these two options may fit together, involving hematopoietic and non‐hematopoietic cells [50]. A few years ago, Tefferi et al. analyzed plasma levels of 30 cytokines in 127 patients with PMF: interleukin (IL)‐8, IL‐2R, IL‐12, IL‐15, and IL‐10 levels were significantly elevated compared to normal controls [51]. In particular, IL‐8 and IL‐2R were associated with transfusion need, leukocytosis, constitutional symptoms, and inferior overall and leukemia‐free survival [51]. Many other studies have investigated cytokine levels in MF patients, although the results were not reproducible across the studies, reflecting differences in disease staging, prior therapies, and technical aspects [52]. Although the full spectrum of inflammatory mediators that play a causal role in MF has yet to be identified, JAK/STAT and nuclear factor κβ (NF‐κβ) inflammatory pathways cooperate to promote fibrosis [53, 54]. More recent studies have highlighted the role of other cytokines such as CXCL4 [55, 56], IL‐6 [57], and IL 1‐β [58].

In addition to megakaryocytes, other cell types play a role in the development and progression of MF. In this regard, a pathological interaction between megakaryocytes and neutrophils has been documented, leading to an anomalous release of cytokines due to an altered distribution of P‐selectin in megakaryocytes [59, 60]. Consequently, neutrophil‐derived enzymes, including elastase, contribute to the pathological process by facilitating the egress of myeloid progenitors into the peripheral blood [61, 62]. Furthermore, the role of monocytes in fibrosis development has been highlighted. BM from patients with PMF revealed an abundance of clonal, neoplastic collagen‐ and fibronectin‐producing monocyte‐derived fibrocytes [63]. Moreover, signaling lymphocytic activation molecule F7 (SLAMF7)+ monocytes were significantly increased compared to controls, correlating with *JAK2* V617F allele burden and serum IL‐1 receptor antagonist [64]. Recently, it has been suggested that regulatory T cells play a central role in the production and activation of TGFβ‐1 and may also limit the immune reaction of CD8 T cells against the neoplastic clone [65]. Mast cells might also be involved in the activation of TGFβ‐1 through the secretion of IL‐13 [66]. Recent studies revealed that glioma‐associated oncogene homolog 1 (Gli1)+ and leptin receptor (LepR)+ mesenchymal stromal cells (MSCs) are progenitors of fibrosis‐causing myofibroblasts in the BM [67, 68], particularly through the expression of the alarmin complex S100A8/S100A9 [69].

Overall, as highlighted above, the pathogenesis of MF is a multifaceted process with complex molecular features and aberrant cellular‐intrinsic and ‐extrinsic inflammatory pathways, characterized by altered cytokine production. Therefore, many different potential targets have been discovered and targeted as the clinical phenotype is highly heterogeneous and difficult to treat.

## 3 Current Targeted Treatments

The discovery of driver mutations and the demonstration of the dependence of MPN on deregulation of the JAK‐STAT pathway have prompted the development of small‐molecule inhibitors of JAK tyrosine kinase (JAKi). These molecules are reversible, orally bioavailable ATP‐competitive inhibitors targeting both wild‐type and mutant JAK2, as well as other JAK family members (JAK1, JAK3 and TYK2) and non‐JAK kinases, resulting in a unique efficacy and safety profile [16].

Ruxolitinib (RUX) is a JAK1 and JAK2 inhibitor that was approved by the Food and Drug Administration (FDA) in 2011 and by the European Medicines Agency (EMA) in 2012; its efficacy and safety were demonstrated in two randomized phase III studies compared to placebo (COMFORT‐I) [70] and best available therapy (BAT, in COMFORT‐II) [71]. Overall, RUX was effective in reducing splenomegaly, symptoms, and improving quality of life in approximately 50% of cases. Spleen response was defined by a reduction ≥ 35% in spleen volume from baseline to week 24 (in COMFORT‐I) and to week 48 (in COMFORT‐II), measured by means of magnetic resonance imaging or computed tomography (SVR ≥ 35%) whereas symptom response was assessed by calculating the proportion of patients with a reduction in the total symptom score ≥ 50% from baseline to week 24, using the modified Myelofibrosis Symptom Assessment Form (TSS ≥ 50%). The single‐arm phase IIIb JUMP trial, including 2233 patients, confirmed the results of the COMFORT studies [72]. The most frequent hematological adverse events (AE) of RUX were dose‐dependent anemia and thrombocytopenia; respective grade 3–4 toxicities were reported in about 40% and 15% of patients. Approximately 10% of patients experienced non‐hematologic AE, including asthenia, fatigue, diarrhea, headache, and infections [73, 74]. Interestingly, recent data suggest the risk of second primary malignancies, particularly non‐melanoma skin cancer (NMSC), possibly due to the immunosuppressive activity of RUX [75, 76]. Despite initial responses, approximately 40%–50% of patients over a median follow‐up of 3 years discontinued the drug due to failure or intolerance, and the median overall survival after RUX discontinuation was 14 months [77, 78]. Discontinuation of ruxolitinib in patients with myelofibrosis is often associated with the acquisition of additional mutations, which contribute to disease progression, resistance to therapy, and poorer overall survival outcomes. In particular, clonal progression and thrombocytopenia at the time of RUX discontinuation were associated with a poor prognosis [77].

Fedratinib (FEDR) is a JAK2/FLT3 inhibitor approved in 2019 by the FDA and in 2021 by the EMA both for JAKi‐naïve patients, as in the JAKARTA study [79], and for RUX refractory/intolerant patients, according to the JAKARTA‐2 study [80, 81]. Anemia and thrombocytopenia were the most common hematologic AEs; notably, grade 3‐4 anemia occurred in 52% and 38% of the JAKARTA and JAKARTA‐2 studies, respectively. The most common non‐hematological AEs were gastrointestinal, including nausea (56%–57%), vomiting (41%–50%), and diarrhea (61%–62%) [79, 80]. According to the JAKARTA study, SVR ≥ 35% and TSS ≥ 50% rates with FEDR in JAKi‐naïve MF were 47% and 40%, respectively [79]. Corresponding rates in RUX‐exposed patients were 30% and 27% (JAKARTA 2) [81]. Response rates were similar in a real‐world setting, including 150 patients who received FEDR following prior RUX failure, with 26.8% of patients achieving ≥ 50% spleen reduction by month 6 [82], while in another study, including patients taking RUX ≥ 20 mg BID before switching to FEDR, spleen response rates were 0% [83]. The risk of Wernicke's encephalopathy, although not clearly related to FEDR [84], is highlighted in a black box warning; therefore, thiamine levels should be checked prior to starting therapy and then periodically.

Neither RUX nor FEDR have been studied in patients with severe thrombocytopenia (< 50 × 10^9^/L) who have more advanced diseases, including anemia, increased risk of bleeding, worse symptom burden, and shorter survival. In this regard, two other less myelosuppressive agents, pacritinib (PAC) and momelotinib (MMB) were approved by the FDA in February 2022 and September 2023, respectively. Except for PAC, all other JAKi have also been approved by the European Medicines Agency (EMA).

Pacritinib (PAC) is a JAK2/FLT3 inhibitor and also inhibits IRAK1, which regulates the synthesis of numerous pro‐inflammatory cytokines, including type I IFN, and activin receptor‐type 1 (ACVR1/ALK2). The latter transduces the bone morphogenic protein (BMP) signal and regulates hepatic hepcidin synthesis [85, 86]. The efficacy of PAC was evaluated in two randomized phase III studies. PERSIST‐1 compared PAC with BAT (excluding RUX) in JAKi‐naïve patients [87]. PERSIST‐2 was developed to evaluate PAC at two different dose levels (200 mg twice daily and 400 mg daily) including those who had previously been exposed to RUX (about 50% of the total). Patients were randomized in a 1:1:1 ratio to PAC 400 mg QD, 200 mg BD, or BAT, including low‐dose RUX [88]. In the PERSIST‐1 study, PAC at 400 mg QD demonstrated a significant and durable reduction in splenomegaly in comparison with BAT. By week 24, SVR ≥ 35% was achieved by 19% and 5% of patients in the PAC and BAT arms, respectively. The difference remained statistically significant across baseline platelet groups, with 23% (PAC) versus 0% (BAT) of patients with severe thrombocytopenia achieving this endpoint. However, the proportions of patients with TSS ≥ 50% at week 24 were similar between the two arms. Of note, among patients who were red blood cell (RBC) transfusion‐dependent (TD) at baseline, 25% of those receiving PAC versus 0% of those on BAT became RBC transfusion independent [87]. At week 24, considering the PERSIST‐2 trial, PAC (combined arms) was superior to BAT for SVR ≥ 35% (18% vs. 3%) but not for TSS ≥ 50%. The PAC203 dose‐finding study in MF patients intolerant of or resistant to RUX confirmed the dose of 200 mg BD as the best, albeit with modest rates of SVR ≥ 35% (9.3%) and TSS ≥ 50% (7.4%) at week 24 [89]. The phase III PACIFICA trial, in which PAC 200 mg twice daily is randomized against physician choice (including low‐dose RUX), is now recruiting MF patients with baseline platelets < 50 × 10^9^/L who are JAKi naïve or have received one JAKi.

Momelotinib (MMB) is a JAK1/2 inhibitor that also targets ACVR1(ALK2). Its efficacy was evaluated in 2 phase III studies; SIMPLIFY‐1 [90] tested the non‐inferiority of MMB against RUX in JAKi‐naïve patients, while SIMPLIFY‐2 [91] tested the efficacy of MMB compared to BAT (RUX in 89% of patients) in patients previously exposed to RUX. In SIMPLIFY‐1 at week 24, 26.5% in the MMB arm and 29% in the RUX arm displayed SVR ≥ 35%, meeting the threshold for non‐inferiority, not achieved for TSS ≥ 50% [90]. Conversely, SIMPLIFY‐2 failed to demonstrate the superiority of MMB in SVR ≥ 35% (7% in MMB vs. 6% in BAT) [91]. Interestingly, both studies confirmed a remarkable clinical activity of MMB on anemia. In SIMPLIFY‐1, at week 24 66.5% of the MF patients on MMB achieved or maintained RBC transfusion independence versus 49.3% on the RUX arm [90]; similarly, in SIMPLIFY‐2, 40% of the MMB‐treated patients did not require RBC transfusions as compared to 27% in the BAT arm over the entire treatment period [91]. The recent blinded, placebo‐controlled, phase III trial MOMENTUM recruited JAKi‐exposed MF patients with splenomegaly who were symptomatic and anemic. Patients were randomly assigned to receive MMB, 200 mg daily, or danazol. In addition to demonstrating MMB superiority over symptoms as the study primary endpoint, all anemia‐related secondary endpoints favored MMB [92].

The main characteristics of clinical trials with JAKi and their results are listed in Table 1.

**TABLE 1:** Overview of pivotal clinical trials with JAK inhibitors in myelofibrosis.

JAK inhibitor	Primary targets	Clinical trial	Study phase	Main inclusion criteria	Treatment arms	Primary and main secondary endpoints	Main results	Therapy related adverse events
Ruxolitinib (RUX)	JAK1 JAK2	COMFORT‐I NCT00952289 Randomized (1:1) PLC controlled	III	Intermediate‐2 or high‐risk disease [per International Prognostic Scoring System], < 10% peripheral blasts, a platelet count ≥ 100 × 109/L, and a palpable splenomegaly with ≥ ≥ 5 cm below the left costal margin	RUX as per label (*n* = 155)/ PLC (*n* = 154)	*Primary endpoint*: SVR ≥ 35% at 24 weeks, assessed by imaging. *Secondary endpoints*: Durability of response, changes in symptom burden (assessed by total symptom score‐TSS), and overall survival.	41.9% of RUX versus 0.7% of PLC had a SVR ≥ 35%. Improvement of ≥ 50% in TSS at 24 weeks in 45.9% of RUX versus 5.3% of PLC	Grade 3/4 anemia and thrombocytopenia (46% and 13%, respectively). *% for all grades AEs* Fatigue (25%); diarrhea (23%); peripheral edema (19%); dyspnea (17%) Infections and non‐melanoma skin cancer
		COMFORT‐II NCT00934544 Randomized to BAT (2:1)	III	as COMFORT‐I	RUX as per label (*n* = 146)/BAT (*n* = 73)	As in COMFORT‐I but at week 48.	28% of RUX vs. 0% of BAT had a SVR ≥ 35% at week 48, and 32% versus 0% at week 24	Grade 3/4 anemia and thrombocytopenia 46% and 19%, respectively. *% for all grades AEs* Diarrhea (36%); peripheral edema (33%); urinary tract infection (25%), pneumonia (13%), herpes zoster reactivation (12%). Non‐melanoma skin cancer (17%)
Fedratinib (FEDR)	JAK1 JAK2 TYK2 JAK3	JAKARTA NCT01437787 PLC controlled Randomized (1:1:1)	III	Intermediate‐2 or high‐risk disease [per dynamic International Prognostic Scoring System], JAKi‐naïve, platelet count ≥ 50 × 10^9^/L, and palpable splenomegaly with ≥ ≥ 5 cm below the left costal margin.	FEDR 400 mg QD (*n* = 96) FEDR 500 mg QD (*n* = 97) PLC (*n* = 96)	*Primary endpoint*: proportion of patients with SVR ≥ 35% from baseline to week 24 and confirmed 4 weeks later. *Secondary endpoints*: Proportion of patients with at ≥ 50% reduction in symptom burden (assessed by total symptom score‐TSS).	Rate of primary endpoint was 36%–40%–1% in FEDR 400 500 mg‐ PLC, respectively Improvement of ≥ 50% in TSS at 24 weeks was 36%–34%–7% in FEDR 400–500 mg‐ PLC, respectively	Overall grade 3/4 anemia and thrombocytopenia: 52% and 22%, respectively *% for all grades AEs* Nausea (57%) vomiting (50%), diarrhea (61%). Infections (40%); mainly, urinary tract infections (8%). Laboratory parameter elevation (ALT, AST, creatinine, amylase, lipase). Suspected Wernicke's encephalopathy (4 cases all in the FEDR 500 mg arm)
		JAKARTA‐2 NCT01523171 Single arm	II	As JAKARTA, but including MF patients previously treated with RUX (both resistant and intolerant)	FEDR 400 mg (*n* = 97)	*Primary endpoint*: proportion of patients with SVR ≥ 35% from baseline to week 24 *Secondary endpoints*: proportion of patients with at ≥ 50% reduction in symptom burden (assessed by total symptom score‐TSS).	The primary end point was achieved in 55% of patients Improvement of ≥ 50% in TSS at 24 weeks was achieved in 26% of patients	Overall grade 3/4 anemia and thrombocytopenia: 38% and 22%, respectively *% for all grades AEs* Nausea (56%) vomiting: (41%); diarrhea: (62%). urinary tract infections (8%). Laboratory parameter elevation as in JAKARTA
Pacritinib (PAC)	JAK2 FLT3 IRAK1 CSF1R ACVR1	PERSIST‐1 NCT01773187 Randomized to BAT (2:1)	III	DIPSS Intermediate or high‐risk, JAKi‐naïve, < 10% peripheral blasts, no exclusion for anemia or thrombocytopenia, and palpable splenomegaly with ≥ 5 cm below the left costal margin.	PAC 400 mg QD (*n* = 220) BAT excluding RUX (*n* = 107)	*Primary endpoint*: proportion of patients with SVR ≥ 35% from baseline to week 24. *Secondary endpoints*: proportion of patients with at ≥ 50% reduction in symptom burden (assessed by total symptom score‐TSS).	SVR ≥ 35% at week 24 was 19% in the PAC arm versus 5% in BAT Improvement of ≥ 50% in TSS was 24.5% in PAC arm versus 6.5% in BAT 90 patients in the BAT group crossed over to PAC at a median of 6.3 months	Overall grade 3/4 anemia and thrombocytopenia: 17% and 12%, respectively *% for all grades AEs* Diarrhea (55%); nausea (27%); vomiting (16%). Bleeding events, including epistaxis, hematoma, and post procedural hemorrhage (grade 3/4: 6%). Cardiac events, including cardiac failure, atrial fibrillation, congestive cardiac failure, QT prolongation, syncope, and pulmonary edema (grade 3/4: 12%)
		PERSIST‐2 NCT02055781 Randomized to PAC 400 mg/QD, PAC 200 mg/BD and BAT (1:1:1)	III	Inclusion criteria as PERSIST‐I but platelet counts < 100 × 10^9^/L and allowing prior JAK inhibitor therapy.	PAC 400 mg QD (*n* = 104) PAC 200 mg BD (*n* = 106) BAT including RUX (*n* = 98)	*Primary endpoint*: proportion of patients with SVR ≥ 35% and 50% or more reduction in TSS from baseline to week 24. *Secondary endpoints*: Compare the efficacy of PAC once daily or twice daily vs. BAT.	At week 24, 27 (18%) patients on PAC (11 once daily and 16 on twice daily) achieved SVR ≥ 35% compared with 2 patients (3%) on BAT. At week 24, 37 patients (25%) on PAC (13 once daily and 24 twice daily) achieved improvement of ≥ 50% in TSS compared with 10 patients (14%) on BAT. Overall, PAC 200 mg twice daily was more effective	Overall grade 3/4 anemia and thrombocytopenia: 24% and 31%, respectively *% for all grades AEs* Diarrhea (58%); nausea (35%), vomiting (20%). Bleeding events, including epistaxis, contusion, petechiae, ecchymosis, hematoma, conjunctival hemorrhage, and purpura (grade 3/4: 10%). Cardiac events, including peripheral edema, cardiac failure, atrial fibrillation, and QT prolongation (grade 3/4: 10%)
		PAC203 NCT03165734 Randomized to PAC 100 mg QD or 100 mg BID or 200 mg BID (1:1:1)	II	As PERSIST‐2 but any platelet counts and RUX failure or intolerance	PAC 100 mg QD (*n* = 55), 100 mg BID (*n* = 55) or 200 mg BID (*n* = 55)	*Primary endpoint*: determine the recommended dose of PAC. *Secondary endpoints*: the dose–response relationship for efficacy and safety.	At week 24, SVR ≥ 35: 9.3% (PAC 200 mg BID) vs. 1.8% (100 mg BID) vs. 0% (100 mg QD) 16.7%, if PAC at 200 mg BID and baseline PLT < 50 × 109/L; improvement of ≥ 50% in TSS similar among arms (around 7.5%)	Grade 3/4 anemia and thrombocytopenia: 20.4% and 33.3% at 200 mg BID, respectively. Common GI toxicity, mostly G1/2 at PAC 200 mg BID. Uncommon cardiac events and bleedings
Momelotinib (MMB)	JAK1 JAK2 ACVR1	SIMPLIFY‐1 NCT01969838 Randomized to RUX (1:1)	III	DIPSS int‐1 (with symptomatic organomegaly/anemia/resistance to non‐JAKi therapies), int‐2, high risk; JAKi naive	MMB 200 mg QD (*n* = 215) RUX as per label (*n* = 217)	*Primary endpoint*: proportion of patients with SVR ≥ 35% from baseline to week 24. *Secondary endpoints*: proportion of patients with ≥ ≥ 50% reduction in symptom burden (assessed by total symptom score‐TSS) and red blood cell transfusion independence.	SVR ≥ 35% in 26.5% MMB vs. 29% RUX at week 24. Improvement of ≥ 50% in the total symptom score in 28.4% MMB versus 42.2% RUX. MMB is associated with a reduced transfusion requirement.	Overall grade 3/4 anemia and thrombocytopenia: 6% and 7%, respectively *% for all grades AEs* Diarrhea (18%); headache (18%); dizziness (15%); nausea (16%); fatigue (15%); peripheral neuropathy (10%). First‐dose AEs (including dizziness, hypotension, flushing, nausea, and headache) were reported in 7% of patients.
		SIMPLIFY‐2 NCT02101268 Randomized to BAT (2:1)	III	DIPSS int‐1 (with symptomatic organomegaly), int‐2, high risk. RUX treatment requiring RBC transfusion or dose adjustment for ≥ grade 3 anemia, thrombocytopenia or bleeding	MMB 200 mg QD (*n* = 104) BAT (*n* = 52)	Primary and secondary end points as in SIMPLIFY‐1 with superiority of MMB versus BAT.	MMB was not superior to BAT for spleen response, but significantly better in improving disease related symptoms and transfusion independence	Overall grade 3/4 anemia and thrombocytopenia: 14% and 7%, respectively *% for all grades AEs* Diarrhea (33%); asthenia (19%); nausea (19%); cough (17%); abdominal pain (15%); dizziness (15%); peripheral neuropathy (11%)
		MOMENTUM NCT04173494 Randomized to danazol (2:1)	III	DIPSS int‐1, int‐2, high risk symptomatic MF; Hb < 10 g/dL, platelet count ≥ 25 × 10^9^/L. Previous exposure to any JAKi	MMB 200 mg QD (*n* = 130) vs. Danazol 600 mg QD (*n* = 65)	*Primary endpoint*: proportion of patients with at ≥ 50% reduction in symptom burden. *Secondary endpoints*: Transfusion independence rate at week 24.	Improvement of ≥ 50% in the total symptom score 25% (MMB) vs. 9% (danazol); SVR ≥ 35% 22% (MMB) vs. 3% (danazol). RBC‐transfusion independence at week 24: 30% (MMB) vs. 20% (danazol).	Overall grade 3/4 anemia and thrombocytopenia: 61% and 28%, respectively. *% for all grades AEs* Diarrhea (22%), nausea (16%), asthenia (13%) (61%), peripheral neuropathy (4%)

Abbreviations: AEs, adverse events; BAT, best available therapy; DIPSS, dynamic international prognostic system; ET, essential thrombocythemia; GI, gastrointestinal; int., intermediate; IPSS, international prognostic system; JAKi, JAK inhibitor; MF, myelofibrosis; PLC, placebo; PLT, platelet count; PMF, primary myelofibrosis; PV, polycythemia vera; RBC, red blood cell; SVR, spleen volume reduction.

## 4 Investigational Treatments

Many studies have focused on developing new monotherapies or combination treatments that exhibit complementary activity or act synergistically with JAKi. New investigational agents could target biological pathways other than JAK/STAT and/or improve the efficacy of JAKi, mainly RUX. The main experimental treatments and the available data recently published will be summarized below, underlining the scientific background. In Table 2, some selected MF therapies in clinical development stratified by target are summarized.

**TABLE 2:** Selected MF therapies in clinical development stratified by target.

Drug	Mechanism of action	Study population and arms	Study phase	Primary endpoint	Clinical trial identifier	Status
*Inhibitors of epigenetic regulator*						
Pelabresib (CPI‐0610)	BET inhibitor	JAKi naïve; blinded study comparing pelabresib and RUX with placebo and RUX	III	SVR≥ 35% at week 24	NCT04603495 (MANIFEST‐2)	Active, not recruiting
ABBV‐744	BET inhibitor	JAKi‐naïve or not; ABBV‐744 ± RUX ± navitoclax	Ib	Safety and tolerability	NCT04454658	Active, not recruiting
BMS986158	BET inhibitor	JAKi‐naïve or not; BMS986158 ± RUX or FEDR	Ib/II	Safety and tolerability	NCT04817007	Active, not recruiting
INCB057643	BET inhibitor	JAKi‐naïve or not; INCB057643 ± RUX	I	Safety and tolerability	NCT04279847	Recruiting
Bomedemstat (IMG‐7289)	LSD1 inhibitor	JAKi‐naïve or not; RUX + bomedemstat	II	Safety and tolerability	NCT05569538	Recruiting
*Inhibitors of signaling*						
Parsaclisib	PI3Kδ inhibitor	JAKi‐naïve; parsaclisib +RUX versus placebo +RUX	III	SVR **≥** 35% at week 24	NCT04551066 (LIMBER 313)	Active, not recruiting
Nuvisertib (TP‐3654)	PIM inhibitor	JAKi‐experienced or RUX‐, FEDR‐ineligible; nuvisertib ± RUX ± MMB	I/II	Safety and tolerability; SVR **≥** 35%	NCT04176198	Recruiting
TL‐895	BTK inhibitor	JAKi ineligible, resistant, relapsed or intolerant; TL895 monotherapy	II	Dose finding	NCT04655118	Recruiting
TL‐895	BTK inhibitor	JAKi‐naïve and RUX suboptimal responders; TL‐895 + RUX	Ib/II	Dose finding; SVR ≥ 35%	NCT05280509	Recruiting
*Telomerase inhibitor*						
Imetelstat	Telomerase inhibitor	JAKi relapsed or refractory; imetelstat versus BAT (excluding JAKi)	III	Overall survival	NCT04576156 (MYF3001)	Recruiting
*Cell cycle inhibitors and apoptosis inducers*						
Abemaciclib	CDK4/6 inhibitor	RUX suboptimal responders; abemaciclib + RUX	I	Dose finding; safety and tolerability	NCT05714072	Recruiting
Navtemadlin	MDM2 inhibitor	JAKi relapsed or refractory; navtemadlin versus BAT (excluding JAKi)	II/III	SVR≥ 35% at week 24	NCT03662126 (BOREAS)	Recruiting
Navtemadlin	MDM2 inhibitor	RUX suboptimal responders; navtemadlin + RUX versus placebo + RUX	III	SVR≥ 35% and TSS≥ 50% at week 24	NCT06479135 (POIESIS)	Recruiting
*Inhibitor of nuclear export*						
Selinexor	XPO1 inhibitor	Relapsed, refractory or intolerant to JAK inhibitors; selinexor monotherapy versus BAT (including JAK inhibitor)	II	SVR≥ 35% at week 24	NCT04562870	Active, not recruiting
Selinexor	XPO1 inhibitor	Relapsed, refractory or intolerant to JAK inhibitors; selinexor monotherapy	II	SVR35% at week 24	NCT03627403 (ESSENTIAL)	Active, not recruiting
Selinexor	XPO1 inhibitor	JAKi‐naïve; selinexor + RUX	I/III	Dose finding, safety and tolerability/SVR≥ 35% and TSS≥ 50% at week 24	NCT04562389 (SENTRY)	Recruiting
Selinexor	XPO1 inhibitor	JAKi‐naïve and moderate thrombocytopenia (platelet 50 to 100 x 10^9^/L); selinexor monotherapy	II	SVR35% at week 24	NCT05980806 (SENTRY‐2)	Recruiting
*Anti‐fibrotic therapy*						
GB2064	LOXL2 inhibitor	Refractory, intolerant or ineligible for a JAK inhibitor	IIa	Safety and tolerability	NCT04679870	Active, not recruiting
*Immunotherapy and direct targeting of mutant* CALR						
VAC85135 + ipilimumab	Anti‐CALR mutated vaccine and anti‐CTLA4	JAKi‐naïve MPN with *CALR* mutation or *JAK2* V617F positive with HLA‐A02:01; VAC85135+ ipilimumab	I	Safety and tolerability	NCT05444530	Active, not recruiting
Mutant CALR‐peptide Based Vaccine	Anti‐CALR mutated vaccine	*CALR* mutated MPN (ET and MF); previous use of JAKi is allowed.	I	Safety and tolerability	NCT05025488	Recruiting
INCA033989	Anti‐CALR mutated IgG1 mAb	*CALR* mutated MPN (ET and MF); previous use of JAKi is allowed if 5 half‐lives or 28 days before the first dose of study treatment; INCA033989 ± RUX	I	Safety and tolerability	NCT05936359	Recruiting

Abbreviations: BET, bromodomain and extraterminal domain; CDK4/6, cyclin‐dependent kinases 4/6; CTLA4, cytotoxic T‐lymphocyte associated protein 4; ET, essential thrombocythemia; FEDR, fedratinib; JAKi, JAK inhibitors; LOXL2, lysyl oxidase homolog 2; mAb, monoclonal antibody; MDM2, mouse double minute 2; MF, myelofibrosis; MMB, momelotinib; NCT, national clinical trial; PI3K, phosphoinositide 3‐kinase; PIM, proviral integration site for Moloney murine leukemia virus; RUX, ruxolitinib; SVR35%, spleen volume reduction ≥ 35%; TSS50%, total symptom score reduction ≥ 50%; XPO1, exportin 1.

### 4.1 Inhibitors of Epigenetic Regulator

The Bromodomain and Extra‐Terminal motif (BET) proteins, first described in 1992 and comprising BRD2, BRD3, BRD4, and BRDT, are distinguished by the presence of conserved BD1 and BD2 sequences at their N‐terminals, as well as an extraterminal (ET) structure at the C‐terminus. They interact at various levels with acetylated histones and transcription factors to induce gene expression such as c‐*MYC* and other downstream genes via NF‐κB and TGF‐β pathways [93]. Kleppe et al. demonstrated in an *MPL*‐mutated mouse model that the association of the JQ‐1 BET inhibitor and RUX was synergistic in reducing serum levels of inflammatory cytokines, disease burden, and marrow fibrosis mainly through inhibition of NF‐κB [54]. Based on preclinical results, pelabresib (CPI‐0610), an oral selective BET inhibitor, was evaluated in patients with MF. The phase II MANIFEST study included 3 arms for MF patients: arm 1 (*n* = 86) pelabresib monotherapy in patients intolerant/refractory or ineligible for RUX; arm 2 (*n* = 86) pelabresib as add‐on/second line in patients with an inadequate response to RUX; arm 3 (*n* = 84), first‐line combination therapy of pelabresib with RUX in JAKi‐naïve patients. In arms 1 and 2, patients were subdivided according to their RBC TD status, which was defined as an average of≥ ≥ 2 units per month over 12 weeks. In arm 3 and in the non‐TD subcohorts of arms 1 and 2, the primary endpoint was ≥ SVR ≥ 35% at 24 weeks, whereas for TD patients, it was the conversion to transfusion independence. Overall, results were modest in arms 1 and 2; in arm 1, 11% and 16% of patients achieved ≥ SVR ≥ 35% and transfusion independence, respectively [94]. Similarly, arm 2 showed that ≥ SVR ≥ 35% at 24 weeks was observed in 20% of cases, whereas conversion to transfusion independence was achieved in 16% of patients in the TD cohort [95]. The best responses come from arm 3; at week 24, 68% of the patients achieved ≥ SVR ≥ 35%. Of note, 24% of patients achieved a mean hemoglobin increase of≥ ≥ 1.5 g/dL from baseline over any 12‐week period without RBC transfusions. Moreover, 28% and 29.5% of patients had ≥ 1 grade improvement in fibrosis and > 25% reduction in *JAK2*V617F allele burden, respectively; the latter was associated with SVR ≥ 35% [96]. Based on the data from arm 3, a double‐blind, randomized phase III MANIFEST‐2 trial is ongoing to evaluate the efficacy and safety of pelabresib plus RUX compared with placebo plus RUX in JAKi‐naïve patients. Of over 430 randomized patients, 59% of study patients were intermediate‐1 risk by DIPSS, 37% harbored high molecular risk mutations, and only 14% were RBC TD, at baseline [97]. SVR ≥ 35% at week 24 was achieved in 66% of patients treated with pelabresib+RUX versus 35% on placebo+RUX (*p* <  0.001). Conversely, ≥ TSS ≥ 50% at week 24 did not significantly differ (52.3% pelabresib + RUX, versus 46.3% placebo + RUX; *p* = 0.22) [97, 98]. Grade 3‐4 anemia and thrombocytopenia were documented in 23%/9% and 36%/6% of patients in the pelabresib+RUX and placebo+RUX arms, respectively [97, 98]. Gastrointestinal AEs including diarrhea, constipation, and nausea were comparable in the two treatment arms, except for dysgeusia, which was more prominent in pelabresib‐treated patients (18% vs. 4%) [97, 98]. Correlative studies demonstrated the reduction of inflammatory cytokines and modest improvements in BM fibrosis (≥ 1 grade; 39% vs. 24%) with pelabresib+RUX compared to placebo + RUX [97, 98]. All the reported efficacy and safety outcomes were confirmed in the week 48 follow‐up; in particular, SVR ≥ 35% and TSS ≥ 50% were reported in 56.5% versus 37.5% and 45% versus 39% of patients in the pelabresib + RUX versus placebo + RUX arms [99].

Other BET inhibitors such as BMS‐986158 [100] and INCB057643 [101] are showing a promising activity both as monotherapy and in addition to JAKi. Preliminary data of the latter, including 37 MF patients in part 1 monotherapy and part 2 add‐on therapy with RUX, were reported. Overall, the most common AEs were thrombocytopenia, nausea, and anemia in 59%, 29.5%, and 27.3%, respectively. At week 24, SVR ≥ 35% was achieved by 3/16 and 3/12 evaluable part 1 and part 2 patients, respectively. Similarly, TSS ≥ 50% was achieved by 5/14 and 6/11 part 1 and part 2 patients, respectively. Moreover, 3/33 had an anemia response, whereas 2/6 patients achieved RBC transfusion independence (both in part 1 study) [102].

Histone lysine‐specific demethylase 1 (LSD1/KDM1A) was first identified in 2004 as an epigenetic enzyme able to demethylate specific lysine residues of histone H3, using FAD as a cofactor. LSD1 is part of a larger protein complex, containing Co‐RE‐1 silencing transcription factor (CoREST), nucleosome remodeling and histone deacetylase (NuRD), or other factors that determine cell‐ and site‐specific chromatin remodeling [103]. Moreover, STAT3 activity is modulated by methylation on lysine (K140) and is one of LSD1 substrates [104]. In MPN, LSD1 is overexpressed in the megakaryocytes, erythroid, and myeloid precursors [105]. In a *JAK2* V617F mouse model, the LSD1 inhibitor IMG‐7289 (bomedemstat) alleviated MPN features by normalizing blood cell counts, reducing spleen volumes, marrow fibrosis, and *JAK2* V617F allele burden, acting in synergy with RUX [106]. Based on the above pre‐clinical data, bomedemstat is the only LSD1 inhibitor evaluated in MF patients; in a phase II study including 89 patients, mostly RUX pre‐treated (83%), spleen volume, symptoms, and marrow fibrosis were reduced to varying degrees. The most common non‐hematologic AEs reported by patients were dysgeusia in 36% and diarrhea in 34% [107]. Another study investigating bomedemstat plus RUX comprising a cohort A (bomedemstat as add‐on therapy) and cohort B (in JAKi naïve) [108] is ongoing; in 40 evaluable patients at week 24, 11 patients (27.5%) had a ≥ TSS ≥ 50% (cohort A 25.9% and cohort B 30.7%) and 7 patients (17.5%) whereas ≥ SVR ≥ 35% was reported in 7 patients (17.5%; cohort A 7.4% and cohort B 38.5%). At Week 24, 20 patients (50%) had stable or improved hemoglobin (cohort A 51.9% and cohort B 46.3%) [109].

Protein Arginine Methyltransferase 5 (PRMT5) methylates histone and non‐histone proteins. PRMT5 is phosphorylated by mutated JAK2, impairing its methylase activity, leading to HSC expansion [110]. PRMT5 inhibition was synergistic with RUX in reversing leukocyte and platelet counts, hepatosplenomegaly, and fibrosis in an *MPL* W515L mouse model of MF [111]. A phase I study of the PRMT5 inhibitor PRT543 in patients with myeloid malignancies, including 12 MF (10/12 RUX exposed) demonstrated a reduction in inflammatory markers and an improvement in symptoms and anemia, particularly in those with spliceosome mutations [112].

### 4.2 Inhibitors of Signaling Molecules

The phosphoinositide 3‐kinases (PI3K) pathway via protein kinase B (PKB or AKT) and mammalian target of rapamycin (mTOR) plays an important role in cell proliferation and survival. In vitro studies and MPN mouse models have shown that pan‐PI3K [113], mTOR [114], and AKT inhibitors [115] have major effects, synergistically with RUX. PI3Kδ is the most expressed PI3K isoform in leukocyte and MF CD34+ cells [116]. The dosing, efficacy, and safety of the add‐on PI3Kδ inhibitor parsaclisib for MF patients with a suboptimal response to RUX were evaluated in a phase II study. In particular, the proportion of patients achieving a ≥ 10% decrease in spleen volume at 12 weeks was 28% for daily‐to‐weekly dosing and 59.5% for all‐daily dosing [117]. In light of these results, two phase III trials of parsaclisib in association with RUX were started. LIMBER‐313 studied the combination of parsaclisib or matching placebo and RUX in JAK inhibitor– naïve patients, whereas LIMBER‐304 evaluated parsaclisib as an add‐on treatment in patients with a suboptimal response to RUX. Recently, the latter was discontinued due to a lack of efficiency in the reduction of spleen volume.

The mitogen‐activated protein kinase (MAPK)/ extracellular signal‐regulated kinase (ERK) pathway plays a key role in cancer development. Gene mutations involving the pathway were shown to impact response to RUX treatment and outcome of MPN patients [42, 118]. Mouse models have shown that ERK inhibition increases the effects of JAK2 inhibition on myeloproliferation, fibrosis, and inflammation by decreasing the level of numerous pro‐inflammatory cytokines and osteopontin [119, 120]. The combination of ERK inhibitor rineterkib and RUX was tested in a phase I/II open platform ADORE study (NCT04097821); unfortunately, the trial was interrupted.

The proviral integration site for moloney murine leukemia virus (PIM) family is composed of serine‐threonine kinases that are the direct transcriptional targets of STAT5. PIM1 is overexpressed in MF CD34+ cells. Inhibition of PIM together with RUX reduces fibrosis by decreasing TGF‐β1 levels [121, 122]. A phase I/II study with PIM inhibitor nuvisertib is ongoing.

### 4.3 Telomerase Inhibitor

Telomerase activity is rapidly dividing tissues, comprising cancers, and is required to maintain telomere length and DNA integrity during cell division, thus providing a rationale for targeting telomerase in patients with MF. Imetelstat, a 13‐mer lipid‐conjugated oligonucleotide, is a first‐in‐class telomerase inhibitor that can selectively promote apoptosis and inhibit the proliferation of CD34+ MF, but not normal cells [123]. Imetelstat was first tested in patients with MF, 48% exposed to JAKi, with complete or partial remission occurring in 7/33 patients (21%), particularly those with *SF3B1* or *U2AF1* splicing mutations [124]. In a phase II study including patients relapsed or refractory to JAK inhibitors, a modest effect on spleen volume and symptoms (at week 24, spleen and symptom response rates were 32.2% and 10.2%) was documented in the 9.4‐mg/kg arm, apparently increasing overall survival [125]. Grade 3–4 anemia and thrombocytopenia were the most common AEs. An ongoing phase III randomized trial (IMpactMF) is enrolling patients with JAK inhibitor relapsed or refractory MF, comparing imetelstat to BAT (excluding JAKi), with overall survival benefit as the primary endpoint. No data are available, and the planned interim analysis is expected in early 2026 [126].

### 4.4 Cell Cycle Inhibitors and Apoptosis Inducers

Cyclin‐dependent kinases 4 and 6 (CDK4/6) are critical mediators of cellular transition from G1 into S phase, by phosphorylation of retinoblastoma (Rb) protein. CDK6 is overexpressed in CD34+ cells from *JAK2* and *MPL* mutated mouse models and patients with MF, and regulates transcription in both kinase‐dependent and ‐independent manners [127]. Furthermore, CDK4/6 inhibitors exert a synergistic therapeutic effect with RUX in mouse models [128]. A phase I study of CDK4/6 inhibitor abemaciclib plus RUX is ongoing.

Aurora A is a member of mitotic serine/threonine kinases, mainly involved in the G2 to M phase transition in the cell cycle. The Aurora A kinase inhibitor alisertib leads to partial differentiation and subsequent apoptosis of malignant megakaryocytes in mouse models of MF and acute megakaryoblastic leukemia, as well as in cells from patients. In addition to eradicating atypical megakaryocytes, alisertib normalized blood counts and reversed bone marrow fibrosis [129, 130]. In a phase I study involving 24 MF patients ineligible for or refractory to JAK inhibitors, alisertib reduced splenomegaly and symptom burden in approximately 30% of patients, normalized megakaryocytes, and reduced fibrosis in 5/7 patients for whom sequential marrows were available [131]. The most common AEs were grade 3 cytopenias in 20% of patients [131].

The B‐cell lymphoma (BCL)‐2 family of proteins regulates the mitochondrial apoptotic cell death process comprising anti‐apoptotic and pro‐apoptotic members. Anti‐apoptotic proteins include BCL‐2, BCL‐extra‐large (BCL‐xL), myeloid cell leukemia (MCL)‐1, and BCL‐w. The pro‐apoptotic members can be categorized as those containing only a BH3 region (e.g., BIM, PUMA), which act as initiators, and those containing four BH domains (BAK, BAX), which act as effectors in carrying out apoptosis [132]. In MF, anti‐apoptotic proteins BCL‐xL and BCL‐2 are overexpressed, giving the cancer cells a survival advantage [133]. Navitoclax is a first‐in‐class, orally bioavailable, BH3‐mimetic drug that binds with high affinity to BCL‐xL and BCL‐2 and has been shown to induce cancer cell apoptosis [134]. In preclinical models, RUX and navitoclax induced synergistic cell killing [135]. Navitoclax was evaluated as an add‐on therapy in the phase II REFINE trial, which included MF patients with suboptimal response to RUX. At week 24, SVR ≥ 35% and TSS ≥ 50% were achieved by 26.5% and 30% of patients, respectively. Of interest, marrow fibrosis improved by 1–2 grades in 33 cases, whereas anemia response was achieved by 64% of evaluable patients [136]. 100% of patients experienced at least one adverse event; the most common grade ≥ 3 adverse events were thrombocytopenia (56%), anemia (32%), and pneumonia (12%) [136]. Consequently, the phase III trial TRANSFORM‐1 (NCT04472598) evaluated navitoclax plus RUX compared with placebo plus RUX in JAKi‐naïve patients, whereas the phase III TRANSFORM‐2 (NCT04468984) enrolled RUX relapsed/refractory MF to navitoclax and RUX combination versus BAT. Although TRANSFORM‐1 met its primary endpoint, with 63.2% of experimental arm patients achieving SVR ≥ 35%, TSS ≥ 50% was not met [137]. Moreover, the incidence of grade 3–4 thrombocytopenia (51% vs. 15%) and neutropenia (38% vs. 4%) was markedly higher with navitoclax/RUX compared to placebo/RUX. Not surprisingly, adverse events, mainly thrombocytopenia, led to RUX reductions or interruptions in 90% and 63% of patients in the navitoclax arm [137]. Accordingly, the navitoclax clinical development program and all related clinical trials were discontinued.

Murine double minute 2 (MDM2) is an E3 ubiquitin ligase that targets tumor suppressor p53 for degradation. In normal cells, activated MDM2 plays a significant role in maintaining p53 at low levels. Inactivating mutations in *TP53* or overexpression of MDM2 can overcome p53‐driven cell killing, conferring a survival advantage [138, 139]. Although inactivating *TP53* mutations are uncommon in MF, p53 activity is often suppressed by constitutive JAK‐STAT signaling in CD34+ cells, leading to MDM2 overexpression [140, 141]. The oral MDM2 inhibitor navtemadlin (KRT‐232) demonstrated a promising activity in a phase II study including MF patients relapsed/refractory to JAKi. In particular, at the dose of 240 mg/QD given on days 1–7/28, 16% and 30% of patients had a SVR ≥ 35% and ≥ TSS ≥ 50%, respectively [142]. SVR ≥ 35% correlated with decreased peripheral CD34+ cell count and TNFα levels, improved fibrosis, and reduction of driver mutation burden [143]. The most frequent grade 2–3 AEs were gastrointestinal and hematologic toxicities. The phase III BOREAS trial is ongoing and evaluates the efficacy and safety of navtemadlin versus BAT for *TP53* wild‐type MF patients relapsed/refractory to JAKi (NCT03662126); patients are randomized 2:1 to receive navtemadlin monotherapy 240 mg once daily (day 1‐7/28‐day cycle) or BAT (JAKi were excluded). At week 24, preliminary data on 183 patients documented that 15% and 24% of patients on navtemadlin and 5% and 12% of patients on BAT achieved SVR ≥ 35% and TSS ≥ 50%, respectively. The most common grade 3–4 AEs were thrombocytopenia (37% vs. 21%), anemia (29% vs. 25%), and neutropenia (24% vs. 12%) followed by diarrhea (5% vs. 2%), nausea (3% vs. 0%), and vomiting (2% vs. 0%) with navtemadlin versus BAT, respectively [144]. Of note, navtemadlin improved biomarkers of disease burden in R/R MF patients, suggestive of anti‐clonal activity and disease modification. In particular, reduction in CD34^+^ cell count, driver mutation burden, and serum inflammatory cytokine levels were significantly correlated with ≥ SVR ≥ 35% at week 24 [145]. The phase III POIESIS study testing navtemadlin as add‐on therapy to RUX is ongoing (NCT06479135).

### 4.5 Inhibitor of Nuclear Export

A short hairpin RNA library screening on a *JAK2* V617F HEL cell line identified a particular sensitivity to the inhibition of the nuclear‐cytoplasmic transport (NCT) machinery. These results were confirmed in primary MF CD34+ cells and in a *JAK2‐mutated* mouse model with a combination of a specific NCT compound (selinexor) and RUX. The effects of inhibiting NCT are presumably related to the accumulation of tumor suppressor proteins, including tp53, p21, and p27 in the nucleus [146]. A phase II study of selinexor monotherapy in patients with MF refractory or intolerant to JAK inhibitors resulted in a robust rate of SVR ≥ 35%, achieved by 4 of 10 patients (40%) at week 24; moreover, there was an improvement in anemia, symptoms, and a reduction in marrow fibrosis. The most common adverse events were grade 2–3 gastrointestinal, followed by hematological toxicities [147]. Phase I of XPORT‐MF‐034 tested selinexor plus RUX in JAK inhibitor– naïve MF with promising results. SVR ≥ 35% at week 24 was achieved by 40% of the 40 mg cohort and 79% of the 60 mg cohort≥, and TSS ≥ 50% was achieved by 10% and 58%, respectively [148]. To date, selinexor at the dose of 60 mg is under investigation as monotherapy in patients with JAK inhibitor–naïve MF and moderate thrombocytopenia in the phase II SENTRY‐2 (NCT05980806) and in combination with RUX in the phase III randomized, placebo‐controlled, SENTRY (NCT04562389) trial.

### 4.6 Anti‐Fibrotic Therapies

In 2014, a small trial including only 3 MF patients was conducted, using an anti‐TGF‐β1 antibody with a response on anemia [149]. Another study with AVID 200, a TGF‐β1/3 ligand trap, was conducted based on promising preclinical data on GATA1‐low mice, which documented reduced marrow fibrosis and increased marrow cellularity [150]. In the first‐in‐human phase I trial, 21 patients with advanced MF (more than 90% RUX‐pretreated) were treated with AVID200, infused on day 1 of a 21‐day cycle at 3 dose levels. AVID200 treatment resulted in spleen size, symptom benefit, and platelet count improvements with no evidence of reduction in marrow fibrosis; grade 3/4 anemia and thrombocytopenia occurred in 28.6% and 19.0% of treated patients, respectively [151].

Pentraxin 2 (PTX‐2), a circulating endogenous regulator of the inflammatory response to tissue injury and fibrosis, is reduced in MF patients. The recombinant form, PRM‐151 (zinpentraxin alfa), has been shown to inhibit in vitro fibrocyte differentiation from MF. Moreover, the treatment of xenograft mice significantly prolonged survival and slowed the development of marrow fibrosis [63]. Accordingly, a phase II trial to explore the efficacy and safety of PRM‐151 in patients with MF was conducted; 27 patients received intravenous PRM‐151 weekly or every 4 weeks as monotherapy or an additional therapy for patients on stable‐dose RUX. Overall, 33% of patients were responders. In particular, 29% and 56% of patients had a SVR ≥ 35% and TSS ≥ 50% at any time, respectively. At week 24, 35% of patients with evaluable bone marrow biopsy demonstrated improvement in fibrotic grade ≥ 1. Serious related adverse events (mainly infections) were reported in 15% of patients [152].

The lysyl oxidase family consists of five members designated lysyl oxidase (LOX), and lysyl oxidase like‐1 to lysyl oxidase like‐4 (LOXL1–LOXL4). LOXL2, an ECM enzyme that catalyzes the cross‐linking of collagen and elastin, is widely expressed in many fibrotic diseases, including MF. Its pharmacological inhibition reduces marrow fibrosis [153]. A phase II trial using an anti‐LOXL2 monoclonal antibody simtuzumab was conducted in MF patients alone or in association with RUX; no clinical benefit was reported at week 24 [154]. There is an ongoing phase II trial using an oral inhibitor of LOXL2, GB2064. In 4 evaluable patients, a reduction in collagen fibrosis was observed at 6 months, while spleen volume and hematological parameters remained stable [155].

### 4.7 Immunotherapy and Direct Targeting of Disease Drivers

Suppression of host antitumor immunity is an important escape mechanism that allows expansion of the malignant hematopoietic clone; therefore, tumor‐mediated immune suppression represents an important therapeutic target for a wide variety of malignancies. In this regard, immune checkpoint inhibitors of the programmed cell death protein 1 (PD‐1) and programmed death‐ligand 1 (PD‐L1) pathway are the best known and most tested therapeutic agents in clinical use [156]. Preclinical studies indicate a rationale for the use of PD‐1 inhibition in patients with MF. PD‐1 and PD‐L1 expression by MPN‐derived CD34+ cells is increased compared with CD34+ cells from healthy donors [157]. However, in a phase II trial, the anti‐PD1 antibody pembrolizumab monotherapy failed to induce clinical responses, so the study closed after the first stage, including 10 patients, was completed [158].

Among driver mutations, *JAK2* and *MPL* generate single‐amino‐acid substitutions in their respective proteins, while *CALR* deletion or insertion mutations result in frameshift mutations that generate a novel mutant C‐terminus different from the wild‐type CALR C‐terminus. Interestingly, peptides derived from the mutant C‐terminus are recognized by peripheral mononuclear cells isolated both from patients with CALR mutant MPN and from healthy donors [159, 160], and T cells isolated from patients with CALR mutant MPN recognize and kill autologous CALR mutant cells. Overall, *CALR* mutations encode tumor‐specific antigens (TSAs) recognized by patient T cells [160]. Therefore, therapeutic cancer vaccination targeting the CALR mutant epitope has been explored as a new treatment option. A first‐in‐human phase I trial, using a 36‐aminoamino‐acid peptide vaccine spanning the novel mutant CALR C‐terminus, has been completed, and the vaccine was found to be safe and tolerable. While 8/10 patients with MPN who received the peptide vaccine showed evidence of T‐cell responses, no patient demonstrated a clinical response [161]. There are several reasons why a mutant CALR‐directed peptide vaccine might not induce an immune response, including (i) major histocompatibility complex (MHC)‐1 having a high affinity for these neo‐epitopes is underrepresented in MPN patients; (ii) inadequate immune stimulation by the adjuvant; (iii) CALR is implicated in the peptide loading on MHC; (iv) there are huge levels of soluble mutated CALR that may inhibit phagocytosis of tumor cells by dendritic cells and suppress the effects of PD‐1 blockade [162, 163, 164]. Two phase I trials of mutant CALR vaccine (NCT05444530 and NCT05025488) are ongoing.

Another strategy focused on immunotherapeutic approaches with the goal of targeting the mutant CALR neo‐epitope. In 2020, Kihara et al. reported the generation of the mouse chimeric monoclonal antibody B3, specifically targeting mutant CALR. In a *CALR* del52 ET mouse model, treatment with B3 reduced platelets in the peripheral blood and numbers of megakaryocytes in the bone marrow of the mice [165]. Later on, Achyutuni and colleagues generated a murine IgG2 raised against the human CALR neoantigen and treated homozygous *CALR* del52 transgenic mice with a transient reduction in platelet count [166]. In 2022, Tvorogov et al. reported the development of the monoclonal antibody IgG2α 4D7 directed against the common sequence encoded by *CALR* mutations. The antibody inhibited proliferation of patient samples with *CALR* but not *JAK2* V617F mutations, and prolonged survival in xenografted bone marrow models of mutant CALR‐dependent myeloproliferation [167]. Another milestone was the generation of the human IgG1 mAb INCA033989, introduced by Reis et al. in a plenary abstract at the 2022 American Society of Hematology (ASH) meeting and recently published [168]. INCA033989 inhibited mutant CALR‐induced MPL signaling in murine Ba/F3 cells but showed no effects on *JAK2* or *MPL* mutated cells. In a mouse model of mutant CALR MPN, treatment with an INCA033989 mouse surrogate antibody effectively prevented the development of thrombocytosis and accumulation of megakaryocytes in the bone marrow. INCA033989 reduced the pathogenic self‐renewal of mutant CALR‐positive disease‐initiating cells in both primary and secondary transplantations, illustrating its disease‐modifying potential [168]. A phase I study of INCA033989 (NCT05936359) administered as a monotherapy or in combination with RUX in participants with *CALR* mutated MPN, is ongoing.

Overall, the specific inhibition of JAK2 V617F is proving more difficult than that of mutated CALR. Due to the frequently acquired resistance to all type I JAKi, CHZ868 [169] and AJ1‐10502 [170] type II JAK inhibitors that bind to the inactive conformation of the kinase domain and occupy the ATP‐binding site and the adjoining hydrophobic pocket were tested in preclinical models. Of interest, AJ1‐10502 showed improved efficacy in comparison to RUX, with a selective effect on *JAK2* V617F cells and an enhanced safety profile compared to CHZ868 [170]. Specific JAK2 V617F allosteric inhibitors theoretically seem to have the more straightforward approach to directly impact the clone. Although progress has been obtained in the structure of JAK2 V617F by mutational approaches and ultrastructural analysis [171, 172], there are many limitations to this approach: (i) the majority of JAK2 inhibitors developed over the past two decades target the tyrosine kinase (TK) domain; yet, structural studies show no difference in the structure of the TK domain in wild‐type versus mutated JAK2; (ii) type I inhibitors induce JAK2 protein accumulation and activation loop phosphorylation, preventing ubiquitination and dephosphorylation, which appears exacerbated in the setting of mutated JAK2; (iii) other kinases such as TYK2 or JAK1 and other adaptor proteins activated by V617F‐mediated cytokine receptor dimerization may not be inhibited by JAK2 TK domain inhibitors. Consequently, there is a major interest in developing inhibitors targeting the pseudokinase (PK) domain instead of the catalytic site [173, 174]. More recently, the potential of chimeric antigen receptor T‐cell (CAR‐T) therapy, a treatment that reprograms immune cells to target and destroy cancer cells, has been explored as a novel approach for MF caused by *CALR* alterations. Building on its success in leukemia and lymphoma, they aim to develop CAR‐T cells specifically designed to recognize and eliminate CALR‐mutated cells, with a focus on their suitability as a treatment option for younger MF patients [175]. Preclinical data on the efficacy of a first‐in‐class CAR‐T therapy targeting mutant CALR using human organoids have been reported [176].

## 5 Future Perspectives and Conclusion

As highlighted in the current review, many new molecules have been evaluated (or are under investigation) as an initial targeted therapy or added salvage therapy in MF, alone or in combination with other drugs, especially RUX. Unfortunately, most of these clinical trials have failed, and to date, we only have the possibility of prescribing JAKi with the goal of reducing splenomegaly and alleviating symptoms. Among the latest approved JAKi, MMB seems to satisfy more unmet clinical needs given its ability to also improve anemia in light of its limited myelosuppressive potential.

The ultimate goal of therapy for MF is to cure or prolong life, which can be achieved through allo‐HSCT while no JAKi has demonstrated any disease‐modifying activity. This is not surprising since they are not specific to the oncogenic driver mutations (in fact they are effective also in *CALR* and *MPL* mutated cases) and that their value is a consequence of non‐specific inhibition of JAK–STAT, resulting in suppression of inflammatory cytokines and myeloproliferation.

There is also a need for a consensus definition of “disease modification” to facilitate updated trial designs that can evaluate such an endpoint. Quietly, all past and ongoing phase II and III trials have as primary and main secondary endpoints spleen (SVR ≥ 35%) and symptom reductions, as was in the pivotal COMFORT trials. In some studies, SVR ≥ 35% has been correlated with a survival benefit and/or improved marrow fibrosis; however, neither these data nor those related to driver gene variant allele frequency reduction are sufficiently robust to be included in the FDA or EMA recommendations. Overall survival is likely to be the best clinical evidence of disease modification, but this impact may take many years to become apparent, creating a considerable barrier to the development of new drugs. For this reason, some surrogate endpoints, such as progression‐free survival and cytopenias, could be suitable short‐term solutions for the design of new clinical trials [177, 178]. Moreover, patient‐reported outcomes and economic considerations should always be considered in the evaluation process of new drugs.

Finally, the recent development of specific therapies for mutant CALR will force new endpoint discussions with the potential to change the treatment landscape.

## Ethics Statement

The authors have nothing to report.

## Consent

The authors have nothing to report.

## Conflicts of Interest

G.G.L. declares speaker's bureau fees from Novartis and GSK. P.G. declares speaker's bureau fees from AbbVie, GSK, AOP Health, and Novartis and advisory board fees from GSK, Incyte, and Novartis.

## Data Availability

The authors have nothing to report.
